# BDNF mRNA Expression in Leukocytes and Frontal Cortex Function in Drug Use Disorder

**DOI:** 10.3389/fpsyt.2020.00469

**Published:** 2020-05-19

**Authors:** Quézia Silva Anders, Leonardo Villaverde Buback Ferreira, Livia Carla de Melo Rodrigues, Ester Miyuki Nakamura-Palacios

**Affiliations:** ^1^Laboratory of Cognitive Sciences and Neuropsychopharmacology, Program of Post-Graduation in Physiological Sciences, Health Sciences Center, Federal University of Espírito Santo, Vitória, Brazil; ^2^Laboratory of Neurotoxicology and Psychopharmacology, Program of Post-Graduation in Physiological Sciences, Health Sciences Center, Federal University of Espírito Santo, Vitória, Brazil

**Keywords:** brain-derived neurotrophic factor, mRNA expression, Frontal Assessment Battery scores, crack-cocaine use disorder, alcohol use disorder

## Abstract

The brain-derived neurotrophic factor (BDNF) is a neurotrophin recognized to play a major role in neuroplastic modifications associated to drug abuse, being involved in various behavioral changes found in drug use disorders, such as drug sensitization, craving and relapses. These neuroplastic changes were shown to affect the prefrontal cortex functions, which can be briefly measured through cognitive tests such as the Frontal Assessment Battery (FAB). In this study we investigated the BDNF mRNA expression in peripheral blood lymphocytes of crack-cocaine use disorder (CUD) and alcohol use disorder (AUD) patients, after drug detoxification treatment, using a real-time PCR approach and examining its association to FAB performance. BDNF mRNA expression was found to be higher by 2.25-fold in CUD patients and by 2-fold in the AUD patients when normalized to controls, and these values were found to be associated with FAB scores. This preliminary study evaluates, for the first time, BDNF mRNA expression in leukocytes and its relationship to FAB scores in crack-cocaine and alcohol use disorder patients.

## Introduction

The use of substances with addictive properties has been associated with various cognitive, behavioral and physiological dysfunctions. One of its hallmarks is the maintenance of the drug seeking behavior despite negative consequences, leading to high rates of relapses throughout an individual's life span (1).

These addictive substances promote the phosphorylation of transcription regulatory proteins, such as cyclic AMP response element binding (CREB) and methyl CpG-binding protein 2 (MeCP2). Once phosphorylated, these proteins activate the transcription of the brain-derived neurotrophic factor (BDNF), which promotes structural changes in neuronal circuits (2, 3).

It has been demonstrated that BDNF plays a major role in neuroplastic modifications associated to drug abuse, being involved in various behavioral changes found in drug use disorders, such as drug sensitization, craving and relapses. Furthermore, BDNF levels in peripheral blood were shown to be correlated to central nervous system concentrations in alcoholic patients (4).

These neuroplastic modifications in reward circuits promoted by long-term drug abuse were shown to affect the prefrontal cortex functions (5). This brain region is primarily responsible for several human cognitive abilities—such as planning, working memory and inhibitory behavior, which can be briefly measured through cognitive tests such as the Frontal Assessment Battery (FAB) (6).

Although several previous studies have correlated BDNF levels to psychiatric disorders, including substance use disorders, this preliminary study is the first to evaluate the BDNF mRNA expression in lymphocytes and its relationship to FAB scores in alcohol and crack-cocaine use disorder patients.

## Material and Methods

### Subjects

All subjects were informed about the purposes of the experiment by the principal investigator and signed a written consent before entering the study.

Ten patients, fitting both the ICD-10 equivalent and DSM-V criteria for Crack-Cocaine Use Disorder (CUD) and twelve patients, fitting both ICD-10 equivalent and DSM-V criteria for alcohol use disorder (AUD), of both genders, were recruited between October 2017 and June 2018 from a public hospital specialized in drug dependence treatment in Espírito Santo State, Brazil. They were all receiving standard treatment provided by the hospital, consisting of psychosocial approaches—conducted by a professional team of psychologists, nurses, social workers and physicians. They had passed the drug detoxification period, were clinically stable and were not using any medications for at least two weeks by the time blood samples were collected for this study.

The control group was constituted by twelve healthy non-addicted and aged-matched subjects of both genders, recruited among workers from the University Hospital from Federal University of Espírito Santo and Hospital of Military Police of Espírito Santo.

The inclusion criteria for this study were: (1) male and female patients over the age of 18 years; (2) fulfilling criteria for crack-cocaine or alcohol dependence according to the ICD-10 Classification of Mental and Behavioral Disorders and the Diagnostic and Statistical Manual of Mental Disorders, fifth edition, as determined by clinical evaluation; (3) in stable clinical condition with no need for emergency care; (4) able to read, write, and speak Portuguese; and (5) showing no severe withdrawal signs or symptoms at baseline.

Furthermore, exclusion criteria included: (1) a condition of intoxication or withdrawal due to a substance other than crack-cocaine and alcohol, (2) any unstable mental or medical disorder that would compromise the execution of the protocol or substance abuse or addiction other than crack-cocaine and alcohol dependence, except nicotine and/or caffeine; (3) diagnosis of epilepsy, convulsions, or delirium tremens during abstinence from crack-cocaine and alcohol.

This study was approved by the Brazilian Institutional Review Board of the Federal University of Espírito Santo (CAAE 19403713.6.0000.5060), Brazil. It was conducted in strict adherence to the Declaration of Helsinki and is in accordance with the ethical standards of the Committee on Human Experimentation of the Federal University of Espírito Santo, ES, Brazil.

### Sociodemographic Data

Sociodemographic data was acquired following a structured interview during the global clinical evaluation, after it was established the subjects' adequacy to the inclusion and exclusion criteria and the informed consent was signed, and before the FAB evaluation.

### The Frontal Assessment Battery (FAB)

This brief instrument was applied at the beginning of the study, during the global clinical evaluation, after proper agreement and signature of the consent form. It evaluates six different domains of executive function: conceptualization, mental flexibility, motor programming, interference sensitivity, inhibitory control and autonomy (7). Each item is scored from zero to three, totaling a sum of eighteen points for the maximum score.

### Experimental Protocol

We collected 5 ml of peripheral blood from the cubital vein of our subjects, which were then disposed in tubes containing ethylenediaminetetraacetic acid (EDTA). The interval between blood collection and the isolation of leukocytes was no longer than 3 h. Total RNA was extracted from leucocytes using the Qiamp Blood Mini Kit ^®^ (Qiagen, Germany), and the degree of purity was determined by spectrophotometry. Aliquots of RNA were submitted to reverse transcription for complementary DNA (cDNA) using RT2 First Strand Kit^®^ (Qiagen, Germany) according to the manufacturer's protocol, in a final volume of 20 μl.

Beta-actin was chosen as the housekeeping gene, due to its constitutive nature—i.e. widespread expression in human cells, and common utilization in gene expression studies. Primers used for amplification of BDNF and beta-actin genes in real time PCR reaction were purchased from Qiagen company primer bank.

Real-time quantitative PCR (RT-PCR) was performed using the ABI PRISM 7500 Sequence Detection Systems^®^ (Applied Biosystems, USA) in combination with SYBR green detection (Qiagen, Germany). The reactions were optimized in a 10 µl reaction volume containing 2 µl of cDNA, 5 µl of RT2 SYBR Green ROX FAST Mastermix^®^ (Qiagen, Germany), 0.4 µl of beta-actin (NM_001101.3, Qiagen, Germany) and BDNF (NM_001709.4, Qiagen, Germany) and 2.6 µl of H2O. The general PCR condition profile was: Taq polymerase activation at 95°C for 10 min, followed by 40 cycles of denaturing at 95°C for 15 s, annealing at 60°C for 1 min, and extension at 95°C for 15 s. After amplification, a melting curve was acquired to determine the optimal PCR conditions.

### Data Analysis

The mean cycle threshold (Ct) for *BDNF* was subtracted from the beta-actin mean Ct in each group—control (CONT), CUD and AUD, yielding a ΔCt result for each one. Next, the ΔCt of the control group was subtracted from the ΔCt of the CUD and AUD groups, yielding ΔΔCt values. Fold-change was then calculated using the following formula:

$$2^{-\Delta CtAdictted}/2^{-\Delta CtControl}$$

### Statistical Analysis

A one-way ANCOVA, considering age and tobacco use as covariates, since these variables could influence gene expression, was used to compare quantitative data among groups considering the proportion of BDNF Ct values over beta-actin Ct values (BDNF/Act) for each subject, converted into a logarithm scale (8).

Besides, the potential association between BDNF gene expression and frontal executive performance was analyzed by means of a multiple regression analysis adjusted by age and schooling, considering that these variables could influence the cognitive performance. SPSS Statistics Base 24.0 (SPSS Inc, USA) and GraphPad Prism 7.0 (GraphPad Software Inc, USA) were employed for statistical analysis and graphic presentations, and a *p* value below 0.05 was considered statistically significant.

## Results

We used RT-PCR to measure mRNA expression levels in human peripheral blood lymphocytes of CUD and AUD patients in comparison with non-addicted controls.

### Socio-Demographic Data

Groups were adequately paired by age and gender, but other socio-demographic characteristics were unequal (**Table 1**). Schooling was found to be significantly different between groups (p <0.05) possibly due to a higher proportion of subjects with a middle school degree in the AUD group when compared to a higher proportion of subjects with a high school degree in the control and CUD groups; employment situation was different between groups (p = 0.001), as a larger proportion of CUD and AUD patients were unemployed and/or were working as freelancers, while a smaller proportion of them was formally employed. Marital status was also different among groups (p <0.01) as a higher proportion of individuals were married or living in common law in the control and AUD groups, whereas CUD patients were mostly single or widowed (**Table 1**). These differences could be expected to be seen in crack-cocaine and alcohol addicted populations due to the important behavioral and social consequences of these disorders. Tobacco use was not different between groups and FAB performance was also equivalent among groups (**Table 1**).

**Table 1:** Socio-demographic characteristics of the healthy non-addicted controls (CONT, n = 12), crack-cocaine use disorder (CUD, n = 10) and alcohol use disorder (AUD, n = 12) patients.

				Groups				
				CONT (n = 12)	CUD (n = 10)	AUD (n = 12)		*p-value*
*Sociodemographic characteristics*								
Age *[average (SD)]*			47.9 (11.2)		42.5 (12.4)	42.3 (9.0)	F(2,33) = 1.0	*0.38*
Gender *n* (*%*)	Male Female		8 (66.7%) 4 (33.3%)		8 (80.0%) 2 (20.0%)	5 (41.7%) 7 (58.3%)	*X_2_ = 3.58*	*0.17*
Years of education *n* (*%*)	<5 >6 <9 >10 <13 >13		2 (16.7%) 3 (25.0%) 1 (8.3%) 6 (50.0%)		0 (0.0%) 2 (20.0%) 3 (30.0%) 5 (50.0%)	2 (16.7%) 0 (0.0%) 8 (66.7%) 2 (16.7%)	*X_2_ = 12.64*	0.049*
Employment situation *n* (*%*)	Formal work Public worker Informal work Unemployed Intermittent job Temporary work Retired		5 (41.7%) 2 (16.7%) 0 (0.0%) 0 (0.0%) 0 (0.0%) 4 (33.3%) 1 (8.3%)		1 (10.0%) 0 (0.0%) 1 (10.0%) 4 (40.0%) 4 (40.0%) 0 (0.0%) 0 (0.0%)	1 (8.3%) 0 (0.0%) 0 (0.0%) 9 (75.0%) 1 (8.3%) 0 (0.0%) 1 (8.3%)	*X_2_ = 33.53*	0.001***
Marital status *n* (*%*)	Single Married or stable union Divorced Widowed Undeclared		2 (16.7%) 10 (83.3%) 0 (0.0%) 0 (0.0%) 0 (0.0%)		3 (33.3%) 1 (10.0%) 2 (22.%) 3 (33.3%) 1 (11.1%)	5 (41.7%) 6 (50.0%) 0 (0.0%) 0 (0.0%) 1 (8.3%)	*X_2_ = 20.3*	0.009**
*Drug use pattern*								
Amount of drug used (daily) Age at the start of drug usage (years)				0 (0.0) –	14.8 (6.9) rocks/day 33.4 (9.1)	23.9 (17.0) drinks/day 16.6 (5.0)		
Tobacco use^a^		Yes No		2 (16.7%) 10 (83.3%)	5 (55.6%) 4 (44.4%)	6 (50.0%) 6 (50.5%)	*X_2_ = 4.15*	*0.126*
*Clinical examination*								
FAB^b^				13.3 (3.1)	14.1 (2.7)	12.1 (2.8)	*F(2.33) = 1.0*	*0.27*

FAB, Frontal Assessment Battery; a = data from one subject in the CUD group was missing, b = data from two subjects in the CUD group were missing and were imputed by means of linear regression. *p <0.05, **p <0.01, *** = 0.001.

### BDNF Gene Expression

**Figure 1:**
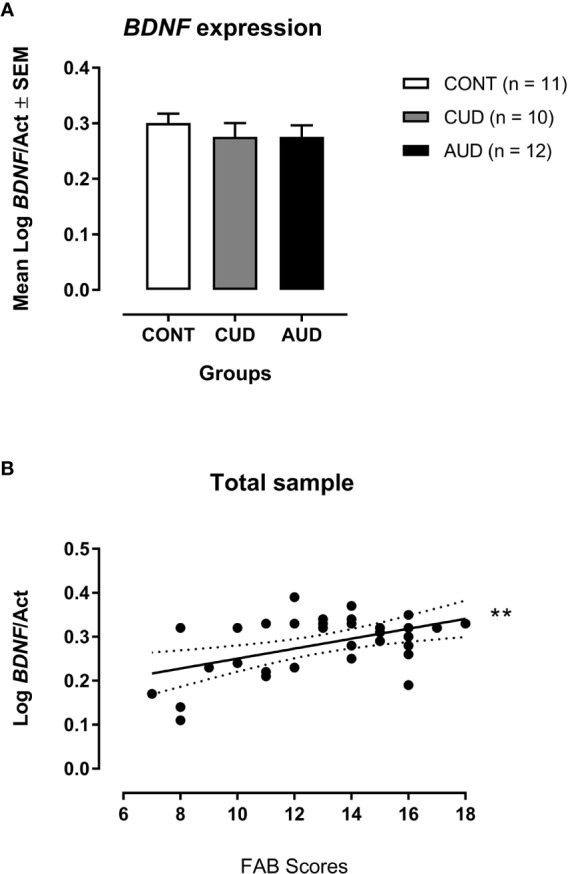
**(A)** Mean Log BDNF/Act values from both CUD (n = 10) and AUD (n = 12) patients compared to the mean of the control group (CONT, n = 11). Smaller Ct values reflect earlier threshold cycle, that is, the quantitative cycle in which the target was captured is early, meaning that the target is more expressed. **(B)** Log BDNF/Act and FAB total scores in all subjects (controls and crack-cocaine and alcohol use disorder patients). **p = 0.002 (Multiple Linear Regression Analysis corrected by age and schooling).

BDNF mRNA expression was found to be higher by 2.25-fold in CUD patients and by 2-fold in AUD patients when normalized to controls (**Table 2**). However, although the Log BDNF/Act was smaller in CUD and AUD groups compared to CONT (**Figure 1A**), no statistically significant difference was found across groups [ANCOVA F(2,32) = 0.78; p = 0.47; R^2^ = 0.054].

**Table 2:** BDNF mRNA expression in control subjects (CONT, n = 11), crack-cocaine use disorder (CUD, n = 10) and alcohol use disorder (AUD, n = 12) patients.

BDNF expression		Groups		
		CONT (n = 11)	CUD (n = 10)	AUD (n = 12)
*Fold change* and Ct values				
Average Ct	*Beta-Actin*	15.66	16.11	17.04
	*BDNF*	32.51	31.85	32.32
ΔCt		32.51 − 15.66 = 16.85	31.85 − 16.11 = 15.74	32.32 − 17.04 = 15.28
*Fold change*			2.25	2.0

### FAB Performance Assessment

Nonetheless, the Hedges' g effect size for two independent samples was 0.36 for CUD (CONT = 0.298 ± 0.051; CUD = 0.270 ± 0.080) and 0.35 for the AUD group (CONT = 0.298 ± 0.051; AUD = 0.276 ± 0.072) when Log BDNF/Act of CUD and AUD groups were compared to CONT separately.

In the multiple regression analysis, with Log BDNF/Act as the independent variable and FAB scores as the dependent variables, including all subjects in this study, Log BDNF/Act, together with age and schooling, accounted for 55.4% of the total FAB scores, F (3,32) = 14.27; p = 0.000007, adjusted R2 = 0.554; 95% CI [−0.008; 8.645]. The Log BDNF/Act showed a significant zero order correlation (r = 0.499, β = 0.303) with FAB scores presenting a significant partial effect (p = 0.002) in the complete model (**Figure 1B**).

## Discussion

Peripheral measurements to detect central nervous system events seem a controversial strategy, even tough, there are studies showing that peripheral leukocytes could be used as biomarkers to measure changes occurring in brain circuits (9, 10).

In gene expression measurements, smaller Ct values reflect earlier threshold cycles, that is, the quantitative cycle in which the target was captured is more precocious, meaning that the target is more expressed (11). In this study, we demonstrated that the Log of Ct values of BDNF/Act was reduced in dependent patients, thus suggesting an increased BDNF mRNA expression in peripheral blood leukocytes of AUD and CUD patients. Despite no statistical significance, the fold change in AUD and CUD groups was twice larger than in the control group. This data agrees with Nubukpo et al. (12), who found a correlation between BDNF gene polymorphisms and increased blood levels of BDNF protein in alcohol use disorder patients.

This increased expression of BDNF mRNA was of a small to medium clinical relevance, according to Cohen's convention (1992), with a Hedges' g effect sizes of 0.36 and 0.35 for CUD and AUD groups, respectively. These results suggest that, irrespectively of statistical significance, the increase in BDNF mRNA expression in patients with substance use disorder can be of clinical importance (13).

Furthermore, we found for the first time an association between BDNF mRNA expression and FAB scores, in which larger logs of BDNF/Act were related to higher FAB scores, thus indicating an inverse relationship between the BDNF mRNA expression and FAB performance—i.e. smaller BDNF mRNA expressions were associated to higher FAB scores, bearing in mind that the log transformation was done over Ct values.

As a neurotrophin, BDNF regulates synaptic plasticity and an increase in its mRNA expression in a condition of long-term drug use could be related to neuroadaptive mechanisms contributing to the excessive dopaminergic function, craving and relapsing behavior, and especially favoring the acquisition of drug-related memories (2, 3, 14). Thus, its increase may reflect a mal adaptative plasticity which could be related to an impairment in executive function.

FAB scale is a brief neuropsychological test which evaluates different prefrontal cortex cognitive domains directly related to executive functions and its scores were negatively related to BDNF mRNA expression. Therefore, BDNF mRNA expression could be a biological marker indicating the severity of executive dysfunctions in patients with substance use disorders (6), a rationale that could be expanded to other conditions impairing prefrontal cortex functions.

There are limitations that must be considered. The high complexity and cost of the method, a limited budget and the restricted inclusion and exclusion criteria utilized, have restricted the number of subjects included in our samples. Moreover, a relative gender imbalance is noted in between our samples, a factor could exert some influence in the results. We have collected 36 samples from non-addicted controls, 27 from AUD and 17 from CUD patients, but included in the analysis only technically adequate samples (i.e. those yielding a minimal total RNA concentration of 25 ng/µl). Here we examined the expression of a single gene, another gene expression study regarding FosB was already published and another one analyzing dopamine receptors will be published soon.

Patients included in this study were recruited from our major clinical trial registered in clinical trials.gov (https://clinicaltrials.gov/ct2/show/NCT02091284 and https://clinicaltrials.gov/ct2/show/NCT02091167).

In summary, in this preliminary study we first showed that the measurements of BDNF mRNA expression in leukocytes from peripheral blood samples can indicate neuronal changes occurring in substance use disorders, and we additionally showed that this measurement may predict frontal executive performance. However, due to the mentioned limitations, more importantly the small sample size, and considering the preliminary nature of this study, more studies are still needed in order to draw more reliable conclusions.

## Data Availability Statement

The datasets generated for this study are available on request to the corresponding author.

## Ethics Statement

The studies involving human participants were reviewed and approved by Brazilian Institutional Review Board of the Federal University of Espírito Santo (CAAE 19403713.6.0000.5060), Brazil. The patients/participants provided their written informed consent to participate in this study.

## Author Contributions

All authors have read and approved the manuscript for submission; have made a substantial contribution to the conception, design, gathering, analysis and/or interpretation of data and a contribution to the writing and intellectual content of the article; and acknowledge that they have exercised due care in ensuring the integrity of the work.

## Funding

EN-P is recipient of a researcher fellowship from Conselho Nacional de Desenvolvimento Científico e Tecnológico (CNPq) (proc. 307531/2018-0) and is also funded by this agency (proc. 466650/2014-0). QA was recipient of graduate student fellowship from Coordenação de Aperfeiçoamento de Pessoal de Nível Superior (CAPES).

## Conflict of Interest

The authors declare that the research was conducted in the absence of any commercial or financial relationships that could be construed as a potential conflict of interest.
